# 

**Published:** 1854-05

**Authors:** 


					﻿
No. Xllj
MAY.
[Vol. IX.
_________j__
BUFFA LO
MEDICAL JOURNAL
AND
MONTHLY REVIEW
or
JEltMtal Surgical Ikicnct.
-
KDITKD BT
AUSTIN FLINT, M. D.
AND
S. B. HUNT, M. D.	t
BUFFALO:
PUBLISHED BY JEWETT, THOMAS A CO.
COMXRBOIAL ADTBRtltn BuiLDINOL
1854
PRICE t2,A0 PER AXNVM.....IN	ADVANCE.
				

